# Molecular landscape of pancreatic cancer: implications for current clinical trials

**DOI:** 10.18632/oncotarget.2972

**Published:** 2015-02-24

**Authors:** Gregory M. Heestand, Razelle Kurzrock

**Affiliations:** ^1^ Center for Personalized Cancer Therapy, Division of Hematology and Oncology, University of California, San Diego, La Jolla, California, 92093, U.S.A

**Keywords:** Pancreatic Cancer, Targeted Therapy, Biomarker Stratification

## Abstract

Despite recent improvements, overall survival for advanced adenocarcinoma of the pancreas continues to be poor. In comparison to other tumor types that have enjoyed marked survival benefit by targeting aberrant cell signaling pathways, standard of care treatment for pancreatic cancer is limited to conventional cytotoxic chemotherapy. Multiple pathway aberrations have been documented in pancreatic cancer. A review of the COSMIC database reveals that most pancreatic cancers contain somatic mutations, with the five most frequent being *KRAS*, *TP53*, *CDKN2A*, *SMAD4*, and *ARID1A*, and multiple other abnormalities seen including, but not limited to, mutations in *STK11/LKB1*, *FBXW7*, *PIK3CA*, and *BRAF*. In the era of tumor profiling, these aberrations may provide an opportunity for new therapeutic approaches. Yet, searching clinicaltrials.gov for recent drug intervention trials for pancreatic adenocarcinoma, remarkably few (10 of 116 (8.6%)) new study protocols registered in the last three years included a molecular/biomarker stratification strategy. Enhanced efforts to target subsets of patients with pancreatic cancer in order to optimize therapy benefit are warranted.

## INTRODUCTION

Pancreatic adenocarcinoma is the fourth most lethal malignancy in the United States, with 39,590 deaths expected in 2014. [1] It is one of the few malignancies where incidence approximates prevalence, as the disease is almost uniformly fatal, often within one year (Table 1). [2, 3] For patients who present with localized disease that can be surgically removed, median survival is 22.8 months due to the high-likelihood of recurrence. [4] Most patients present with metastatic disease, and treatment options are limited to chemotherapy. Median survival is about five to seven months with single-agent gemcitabine, or 8.5–11 months with more intense regimens such as gemcitabine/nab-paclitaxel and fluorouracil (5-FU)/irinotecan/oxaliplatin (FOLFIRINOX). [5–7]

**Table 1:** Current treatment strategies for newly-diagnosed pancreatic cancer

Clinical Scenario	Treatment	MedianOS	1-year Survival	Comment	Reference(s)
Resectable	Surgical Resection; 6 Months Adjuvant Therapy with Gemcitabine and 5-FU Chemoradiation	20.5 Mos	73%	Pancreas head lesions only	Regine et al, 2011 [55]
	Surgical Resection; 6 Months Adjuvant Gemcitabine	22.8 Mos	72%		Oettle et al, 2007, 2013 [4, 56]
Locally-Advanced	3 Months Chemotherapy; 5-FU Chemoradiation	15.0 Mos	65%	Patients that developed mets post-chemo were excluded	Huguet at al, 2007 [57]
Metastatic	FOLFIRINOX	11.1 Mos	48%		Conroy et al, 2011 [7]
	Gemcitabine + Nab-paclitaxel	8.5 Mos	35%		Von Hoff et al, 2013 [6]
	Gemcitabine	5.7 Mos	18%		Burris et al, 1997 [5]

Recent survival gains in the treatment of pancreatic cancer have resulted from new combinations of conventional, non-targeted chemotherapies, such as FOLFIRINOX. [7] Only one targeted agent – erlotinib, a small-molecule tyrosine-kinase inhibitor of EGFR – has been shown to improve overall survival (OS) when combined with gemcitabine. [8] This improvement was a modest 11 days compared to gemcitabine alone. Other targeted agents, such as bevacizumab, cetuximab, and sorefenib, did not improve overall survival in combination with gemcitabine. [9–11] Trials with these agents were open to all patients with pancreatic cancer, and there were no screening criteria to select patients most likely to respond to the targeted agents.

Outside the field of pancreatic cancer, significant advances in oncology therapy have emerged by identifying and intervening upon “actionable” aberrations. Advanced melanoma, which traditionally held a grave prognosis, has undergone a renaissance in treatment options. For the approximately 40% of patients that harbor a V600E *BRAF* mutation, vemurafenib produces a response rate of 48%. [12, 13] Dabrafenib, another BRAF inhibitor, and trametinib, a MEK inhibitor, have also substantially improved outcomes in *BRAF*-mutant patients. [14, 15] In advanced non-small cell lung cancer (NSCLC), median survival with traditional platinum-doublet chemotherapy is eight to nine months. [16] Targeting the EML4-ALK fusion product (~5% incidence) with crizotinib, a small-molecule kinase inhibitor, is associated with a survival of about 20 months. [17] Even erlotinib, whose effect is modest in pancreatic cancer, can improve first-line progression-free survival (PFS) in patients with *EGFR*-mutations (about 8–30% depending upon ethnicity) from 4.6 to 13 months. [18–20] Further, a meta-analysis of the NSCLC literature showed responses of 49% when targeted treatments were matched to the appropriate subgroup of patients, but only 9.7% when unselected populations were treated. [21]

In this regard, a review of pancreas tumor specimens within the Catalogue of Somatic Mutations in Cancer (COSMIC) database reveals that most pancreatic cancers harbor somatic mutations (Table 2), with the five most frequent aberrations being *KRAS*, *TP53*, *CDKN2A*, *SMAD4*, and *ARID1A*. [22, 23] If specific “actionable” mutations can drive marked improvements in survival in melanoma and NSCLC, similar opportunities might reasonably be expected in pancreatic cancer. Herein we review the molecular landscape in pancreatic cancer and provide an overview of the status of current clinical trials in the field.

**Table 2:** Most common mutated genes of pancreatic ductal carcinoma in COSMIC database*

Mutated Gene	Frequency		Wild-Type Gene Function**
	Percentage	Denominator	
*KRAS*	71%	4573	GTPase mediating cell signaling
*TP53*	49%	796	Tumor suppressor
*CDKN2A*	22%	950	Tumor suppressor
*SMAD4*	20%	680	Signal transduction protein
*ARID1A*	6%	343	Chromatin remodeling
*MLL3*	4%	292	Histone methylation
*PIK3CA*	3%	377	Protein kinase mediating cell signaling
*MAP2K4*	3%	294	Protein kinase mediating cell signaling
*ATM*	3%	190	Protein kinase mediating cell cycle checkpoint signaling
*ACVR1B*	3%	226	Growth factor receptor kinase
*BRAF*	2%	528	Protein kinase mediating cell signaling
*APC*	2%	267	Tumor suppressor
*SF3B1*	2%	206	RNA splicing
*STK11/LKB1*	2%	314	Tumor suppressor
*FBXW7*	2%	242	Component of SCF-complex mediating ubiquitination
*SMARCA4*	1%	291	Transcriptional regulation
*ARID2*	2%	189	Transcriptional regulation
*CREBBP*	2%	190	Histone acetylation
*RNF43*	2%	197	Ubiquitin ligase
*EP300*	1%	201	Histone acetylation
ERBB2***	0.4%	256	Receptor tyrosine kinase

* Accessed December 1, 2014 (http://www.sanger.ac.uk/cosmic) [22].

** National Center for Biotechnology Information (NCBI), U.S. National Library of Medicine, Gene database (http://www.ncbi.nlm.nih.gov/gene).

*** In addition to mutation, HER2 may be overexpressed or amplified in ~10 to 30% of patients [58–60].

### KRAS

Seventy-one percent of pancreatic cancer specimens in the COSMIC database harbor *KRAS* mutations. [22] KRAS is a key protein in multiple signaling pathways. When bound to guanosine triphosphate (GTP), it mediates cell survival and differentiation. Common *KRAS* mutations hinder its ability to hydrolyze GTP, leaving it constitutively active. [24] *KRAS* mutations are common in pancreatic duct lesions and are thought to play an early role in oncogenesis. [25] Thus, blocking targets downstream of KRAS is of clinical interest.

One key target downstream of KRAS is MEK, which functions as a protein kinase. Multiple MEK inhibitors are in development, and some have shown promise (Table 3). Selumetinib, a small-molecule MEK inhibitor, was randomized against single-agent capecitabine as a second-line treatment for advanced pancreatic cancer. Though there was no difference in overall survival, two of 38 (5.2%) patients in the selumetinib arm achieved a partial response (PR) [26]. Trametinib has also shown some activity. In a treatment-refractory phase I population, two of 26 patients (8%) achieved PR [27]. However, in a randomized phase II trial with trametinib given in combination with gemcitabine versus gemcitabine alone, response rate was 22% (but included one complete remission) as compared to 18%; survival was 8.4 versus 6.7 months (p, not significant). [28] The fact that some patients respond to MEK inhibitors alone is of interest, though combinations of MEK inhibitors with gemcitabine do not substantially increase the response rate. Whether or not MEK inhibitors in combination with other regimens such as FOLFIRINOX might be beneficial merits further study.

**Table 3:** Clinical trials with MEK1/2 inhibitors in metastatic pancreatic cancer

Agent	Line of Therapy	Phase of Study	Partial Remission (PR)	Comment	Reference
Trametinib + Gemcitabine	1^st^ Line	Randomized Phase II	17/77 (22%);(includes one complete remission	PR 14/77 (18%) in placebo + gemcitabine arm	Infante et al, 2014 [28]
Trametinib + Gemcitabine	Mixed	Phase Ib	3/11 (27%)	Two patients had received prior therapy	Infante et al, 2013 [61]
Trametinib	Refractory	Phase I	2/26 (8%)		Infante et al, 2012 [27]
Selumetinib vs. Capecitabine	2nd Line	Phase II	2/38 (5%)		Bodoky et al, 2011 [26]
XL-518 / GDC-0973 + GDC-0941 (PI3K)	Refractory	Phase Ib	1 PR	Patient with PR had a *BRAF* mutation	LoRusso et al, 2012 [62]
CI-1040	1st Line	Phase II	0/15 (0%)		Rinehart et al, 2004 [63]
CI-1040	Refractory	Phase I	1/6 (17%)		LoRusso et al, 2005 [64]

### KRAS

Forty-nine percent of pancreatic cancers in the COSMIC database demonstrate *TP53* mutations. [22] p53 is key tumor suppressor, and when in an inactivated state, enables cancerous cells to avoid apoptosis. Wee-1 inhibitors such as MK1775 target aberrant p53 by blocking cell cycle checkpoint regulation and increasing susceptibility to cytotoxic chemotherapy. [29] In addition, retrospective analysis by Said et al [30] suggested that tumors with aberrant p53 may be more sensitive to bevacizumab. Patients with aberrant p53 had a median PFS of 11 months while the median PFS in those with wild-type p53 was 5.0 months. On multivariate analysis, the interaction between p53 mutation status and bevacizumab therapy was statistically significant [HR 0.15, 95% CI 0.05–0.44, *p* < 0.001]. [30]

Of additional clinical interest is re-activating p53 in wild-type patients. MDM2, an inhibitor of p53, is overexpressed in many cancers. [31] MDM2–p53 interaction prompts p53 degradation and blocks its tumor suppressor function. [31] Blocking MDM2 activity may prevent this degradation, thereby enabling p53-induced apoptosis of cancerous cells. [32] A search of clinicaltrials.gov lists multiple MDM2 antagonists currently under early-phase investigation, including RO5045337, RO5503781, and DS-3032b. [33]

### KRAS

*CDKN2A* is aberrant in twenty-two percent of patients with pancreatic cancer. [22] It encodes multiple proteins which play roles in tumor suppression. Two transcripts, p16 and p14^ARF^, are frequently abnormal in pancreatic cancer and result in loss of function. p16 inhibits the activity of cyclin-dependent kinases 4/6, thereby playing a regulatory role in the cell cycle by preventing phosphorylation of the tumor suppressor retinoblastoma protein. [34] Loss of p16 results in activation of CDK4/6 and is associated with high-grade pre-malignant pancreatic lesions. [35] Palbociclib, an inhibitor of CDK4/6, has been shown to suppress growth of pancreatic cancer cell lines, though with upregulation of genes associated with metastasis. [36] p14^ARF^ is an inhibitor of MDM2 and stabilizes retinoblastoma protein by interfering with MDM2-mediated degradation. [37] Theoretically, either CDK4/6 or MDM2 inhibitors might be active in patients with loss of *CDKN2A* function.

### KRAS

SMAD4 is a co-factor that facilitates gene transcription and tumor suppression through the TGF-beta signaling pathway. *SMAD4* mutations are present in twenty percent of pancreatic cancers and have been associated a poorer prognosis and increased metastases. [22, 38, 39] Inactivation of *SMAD4* may enable TGF-beta signaling, which is usually suppressive, to promote cancer growth. [40, 41] To our knowledge, the role of TGF-beta inhibitors in patients with SMAD4 mutations has not been investigated.

### KRAS

*ARID1a* mutations are present in six percent of pancreatic cancers. [22] ARID1a plays a role in chromatin remodeling, is thought to have tumor suppressor function, and binds p53. [42] It also modulates signaling through the PI3K/AKT/mTOR axis. [43] Whether or not mutations in *ARID1a* can be targeted by using PIK3CA, AKT or mTOR inhibitors is currently unknown.

### Other potentially actionable mutations

BRCA2 is a potent tumor suppressor and plays a key role in DNA repair. Murphy et al demonstrated that 5/29 patients (17%) with a strong family history of pancreatic cancer harbored *BRCA2* mutations. [44] PALB2, which binds BRCA2, also plays a role in DNA repair. *PALB2* mutations were reported in 3/96 patients (3.1%) with familial pancreatic cancer. [45]

In theory, patients with *BRCA2* or *PALB2* mutations should be more sensitive to DNA-damaging agents. Two case reports illustrate this point (Table 4). Villarroel et al reported a 61-year-old man with metastatic pancreatic cancer in the setting of a *PALB2* mutation. [46] He was initially treated with gemcitabine chemotherapy (nucleoside analogue) with no response, but then received mitomycin C chemotherapy (DNA crosslinker) and achieved a partial response that lasted twenty-two months. A 49-year-old woman with advanced pancreatic cancer in the setting of *BRCA2* mutation who was treated with mitomycin C and capecitabine after progressing through two previous regimens also achieved a partial response. [47] Mitomycin C was discontinued after six months due to toxicity. Patients with *BRCA2* mutations may also be sensitive to PARP inhibitors. Response has been seen in a variety of other tumors, including breast, prostate, and ovarian cancer harboring *BRCA2* aberrations. [48]

**Table 4:** Case reports of novel therapies in advanced pancreatic cancer

Aberration	Histology	Agent	Line	Outcome	Rationale for Agent	Reference
*PALB2* Mutation	Adenocarcinoma	Mitomycin C	2nd	Partial Response	Patient's tumor xenograft demonstrated sensitivity to mitomycin C	Villarroel et al, 2011 [46]
*BRCA2* Mutation	Adenocarcinoma	Mitomycin C + Capecitabine	3rd	Partial Response	Pre-clinical data with mitomycin C in *BRCA2* cell lines and prior published responses to mitomycin C	Chalasani et al, 2008 [47]
*STK11/LKB1* Mutation	Acinar Cell Carcioma	Everolimus	1st	Partial Response	Loss of mTOR inhibition with *STK11/LKB1* mutation	Klumpen et al, 2011 [50]

Abberations in *STK11/LKB1* and *FBXW7* are also potential targets. LKB1 acts through AMPK to inhibit mTOR, which regulates cell growth. [49] Germline *STK11/LKB1* loss-of function mutations are associated with Peutz-Jeghers Syndrome, which carries an increased risk for pancreatic neoplasms. [49] A case reported by Klumpen at al successfully used the mTOR inhibitor everolimus in a Peutz-Jeghers Syndrome patient with pancreatic cancer to obtain a partial response without additional cytotoxic chemotherapy (Table 4). [50] FBXW7 plays a role in the ubiquitin-mediated degradation of oncoproteins, and among patients with NSCLC, low *FBXW7* expression is associated with decreased survival and taxane resistance. In NSCLC cell lines with silenced *FBXW7*, taxane sensitivity can be restored when treated with the histone deacetylase inhibitor MS-275. [51]

Other potentially actionable aberrations that can be seen in small, but not insignificant subsets of patients include *PIK3CA* and *BRAF* mutations. These abnormalities occur in 2 to 3% of patients, and can theoretically be targeted by PI3K/AKT/mTOR and BRAF or MEK inhibitors, respectively.

### Clinical trials for pancreatic cancer

A search of clinicaltrials.gov for new pancreatic cancer protocols registered during the past three years identified 314 protocols (search criteria: trials registered in database during the period 03/01/2011 to 03/01/2014; pancreatic cancer; drug or biological intervention studies). [33] The 314 protocol summaries were manually reviewed, and protocols containing external radiotherapy, neuroendocrine histology, and local therapy were excluded, as were protocols without the stated outcome of improved OS, PFS, or radiographic/biochemical response, leaving only 116 specific for pancreatic adenocarcinoma systemic therapy. Of these 116 protocols, 10 (8.6%) used selective inclusion criteria to identify a subset of patients based upon laboratory molecular/biomarker data and treat with a cognate therapy that was believed to impact or biologically match the biomarker. Six used tissue biomarkers to assign patients among multiple conventional chemotherapy options, two sought patients with *BRCA* mutations for PARP inhibitors, one required a specific antigen for a corresponding investigational monoclonal antibody, and one sought patients with a specific HLA marker for a vaccine study. These results suggest that there is still a remarkable paucity of trials addressing molecular/biomarker stratification in pancreatic cancer.

## CONCLUSIONS

The outcome for patients diagnosed with pancreatic cancer is grim, with one year survival of 19% and 4.8% alive at five years. [52] The best therapy to date is FOLFIRINOX, and it improves median survival by four months (from 6.8 to 11.1 months) compared to gemcitabine for metastatic disease. [7] In other tumor types such as *BRAF*-aberrant melanoma, or *EGFR*- or *ALK*-aberrant NSCLC, significant improvements have been achieved by matching targeted agents with patients harboring the cognate molecular abnormality. Several theoretically “actionable” aberrations exist in pancreatic cancer including, but not limited to, *KRAS, CDKN2A, ARID1A, BRCA, PALB2, PIK3CA, BRAF* and so forth. Despite the number of aberrations that can be targeted, relatively few have been addressed in clinical trials of pancreatic cancer, with only about 9% of clinical trials of pancreatic cancer stratified by a biomarker in the last three years. Although an important step in pancreatic cancer, as in *EGFR*- or *ALK*-mutant lung cancer or BRAF-mutant melanoma might include investigation of matched targeted monotherapy, many pancreatic tumors likely contain more than one aberration. If two or more genomic aberrations exist, the role of each might need to be ascertained, and each important driver may need to be targeted (customized combination therapy) in order to prevent or circumvent resistance. [53, 54] Taken together, the data suggests that efforts to target biomarker-defined subsets of patients with pancreatic cancer in order to optimize therapy benefit are warranted.

## References

[1] Siegel R, Ma J, Zou Z, Jemal A (2014). Cancer statistics, 2014. CA: a cancer journal for clinicians.

[2] Howlader N NA, Krapcho M, Garshell J, Neyman N, Altekruse SF, Kosary CL, Yu M, Ruhl J, Tatalovich Z, Cho H, Mariotto A, Lewis DR, Chen HS, Feuer EJ, Cronin KA SEER Cancer Statistics Review, 1975–2010.

[3] Jemal A, Siegel R, Xu J, Ward E (2010). Cancer statistics, 2010. CA: a cancer journal for clinicians.

[4] Oettle H, Neuhaus P, Hochhaus A, Hartmann JT, Gellert K, Ridwelski K, Niedergethmann M, Zulke C, Fahlke J, Arning MB, Sinn M, Hinke A, Riess H (2013). Adjuvant chemotherapy with gemcitabine and long-term outcomes among patients with resected pancreatic cancer: the CONKO-001 randomized trial. JAMA : the journal of the American Medical Association.

[5] Burris HA, Moore MJ, Andersen J, Green MR, Rothenberg ML, Modiano MR, Cripps MC, Portenoy RK, Storniolo AM, Tarassoff P, Nelson R, Dorr FA, Stephens CD, Von Hoff DD (1997). Improvements in survival and clinical benefit with gemcitabine as first-line therapy for patients with advanced pancreas cancer: a randomized trial. Journal of clinical oncology : official journal of the American Society of Clinical Oncology.

[6] Von Hoff DD, Ervin T, Arena FP, Chiorean EG, Infante J, Moore M, Seay T, Tjulandin SA, Ma WW, Saleh MN, Harris M, Reni M, Dowden S, Laheru D, Bahary N, Ramanathan RK (2013). Increased survival in pancreatic cancer with nab-paclitaxel plus gemcitabine. The New England journal of medicine.

[7] Conroy T, Desseigne F, Ychou M, Bouche O, Guimbaud R, Becouarn Y, Adenis A, Raoul JL, Gourgou-Bourgade S, de la Fouchardiere C, Bennouna J, Bachet JB, Khemissa-Akouz F, Pere-Verge D, Delbaldo C, Assenat E (2011). FOLFIRINOX versus gemcitabine for metastatic pancreatic cancer. The New England journal of medicine.

[8] Moore MJ, Goldstein D, Hamm J, Figer A, Hecht JR, Gallinger S, Au HJ, Murawa P, Walde D, Wolff RA, Campos D, Lim R, Ding K, Clark G, Voskoglou-Nomikos T, Ptasynski M (2007). Erlotinib plus gemcitabine compared with gemcitabine alone in patients with advanced pancreatic cancer: a phase III trial of the National Cancer Institute of Canada Clinical Trials Group. Journal of clinical oncology : official journal of the American Society of Clinical Oncology.

[9] Kindler HL, Niedzwiecki D, Hollis D, Sutherland S, Schrag D, Hurwitz H, Innocenti F, Mulcahy MF, O'Reilly E, Wozniak TF, Picus J, Bhargava P, Mayer RJ, Schilsky RL, Goldberg RM (2010). Gemcitabine plus bevacizumab compared with gemcitabine plus placebo in patients with advanced pancreatic cancer: phase III trial of the Cancer and Leukemia Group B (CALGB 80303). Journal of clinical oncology : official journal of the American Society of Clinical Oncology.

[10] Philip PA, Benedetti J, Corless CL, Wong R, O'Reilly EM, Flynn PJ, Rowland KM, Atkins JN, Mirtsching BC, Rivkin SE, Khorana AA, Goldman B, Fenoglio-Preiser CM, Abbruzzese JL, Blanke CD (2010). Phase III study comparing gemcitabine plus cetuximab versus gemcitabine in patients with advanced pancreatic adenocarcinoma: Southwest Oncology Group-directed intergroup trial S0205. Journal of clinical oncology : official journal of the American Society of Clinical Oncology.

[11] Goncalves A, Gilabert M, Francois E, Dahan L, Perrier H, Lamy R, Re D, Largillier R, Gasmi M, Tchiknavorian X, Esterni B, Genre D, Moureau-Zabotto L, Giovannini M, Seitz JF, Delpero JR (2012). BAYPAN study: a double-blind phase III randomized trial comparing gemcitabine plus sorafenib and gemcitabine plus placebo in patients with advanced pancreatic cancer. Annals of oncology : official journal of the European Society for Medical Oncology/ESMO.

[12] Chapman PB, Hauschild A, Robert C, Larkin JMG, Haanen JBAG, Ribas A, Hogg D, Hamid O, Ascierto PA, Testori A, Lorigan P, Dummer R, Sosman JA, Garbe C, Maio M, Nolop KB (2012). Updated overall survival (OS) results for BRIM-3, a phase III randomized, open-label, multicenter trial comparing BRAF inhibitor vemurafenib (vem) with dacarbazine (DTIC) in previously untreated patients with BRAFV600E-mutated melanoma. ASCO Meeting Abstracts.

[13] Ascierto PA, Kirkwood JM, Grob JJ, Simeone E, Grimaldi AM, Maio M, Palmieri G, Testori A, Marincola FM, Mozzillo N (2012). The role of BRAF V600 mutation in melanoma. Journal of translational medicine.

[14] Falchook GS, Long GV, Kurzrock R, Kim KB, Arkenau TH, Brown MP, Hamid O, Infante JR, Millward M, Pavlick AC, O'Day SJ, Blackman SC, Curtis CM, Lebowitz P, Ma B, Ouellet D (2012). Dabrafenib in patients with melanoma, untreated brain metastases, and other solid tumours: a phase 1 dose-escalation trial. Lancet.

[15] Falchook GS, Lewis KD, Infante JR, Gordon MS, Vogelzang NJ, DeMarini DJ, Sun P, Moy C, Szabo SA, Roadcap LT, Peddareddigari VG, Lebowitz PF, Le NT, Burris HA, Messersmith WA, O'Dwyer PJ (2012). Activity of the oral MEK inhibitor trametinib in patients with advanced melanoma: a phase 1 dose-escalation trial. The lancet oncology.

[16] Ardizzoni A, Boni L, Tiseo M, Fossella FV, Schiller JH, Paesmans M, Radosavljevic D, Paccagnella A, Zatloukal P, Mazzanti P, Bisset D, Rosell R (2007). Group CM-a: Cisplatin-versus carboplatin-based chemotherapy in first-line treatment of advanced non-small-cell lung cancer: an individual patient data meta-analysis. Journal of the National Cancer Institute.

[17] Shaw AT, Kim DW, Nakagawa K, Seto T, Crino L, Ahn MJ, De Pas T, Besse B, Solomon BJ, Blackhall F, Wu YL, Thomas M, O'Byrne KJ, Moro-Sibilot D, Camidge DR, Mok T (2013). Crizotinib versus chemotherapy in advanced ALK-positive lung cancer. The New England journal of medicine.

[18] Zhou C, Wu YL, Chen G, Feng J, Liu XQ, Wang C, Zhang S, Wang J, Zhou S, Ren S, Lu S, Zhang L, Hu C, Hu C, Luo Y, Chen L (2011). Erlotinib versus chemotherapy as first-line treatment for patients with advanced EGFR mutation-positive non-small-cell lung cancer (OPTIMAL, CTONG-0802): a multicentre, open-label, randomised, phase 3 study. The lancet oncology.

[19] Rosell R, Moran T, Queralt C, Porta R, Cardenal F, Camps C, Majem M, Lopez-Vivanco G, Isla D, Provencio M, Insa A, Massuti B, Gonzalez-Larriba JL, Paz-Ares L, Bover I, Garcia-Campelo R (2009). Screening for epidermal growth factor receptor mutations in lung cancer. The New England journal of medicine.

[20] Shigematsu H, Lin L, Takahashi T, Nomura M, Suzuki M, Wistuba II, Fong KM, Lee H, Toyooka S, Shimizu N, Fujisawa T, Feng Z, Roth JA, Herz J, Minna JD, Gazdar AF (2005). Clinical and biological features associated with epidermal growth factor receptor gene mutations in lung cancers. Journal of the National Cancer Institute.

[21] Janku F, Berry DA, Gong J, Parsons HA, Stewart DJ, Kurzrock R (2012). Outcomes of phase II clinical trials with single-agent therapies in advanced/metastatic non-small cell lung cancer published between 2000 and 2009. Clinical cancer research : an official journal of the American Association for Cancer Research.

[22] Forbes SA, Bindal N, Bamford S, Cole C, Kok CY, Beare D, Jia M, Shepherd R, Leung K, Menzies A, Teague JW, Campbell PJ, Stratton MR, Futreal PA (2011). COSMIC: mining complete cancer genomes in the Catalogue of Somatic Mutations in Cancer. Nucleic acids research.

[23] Catalogue of Somatic Mutations in Cancer (COSMIC) http://www.sanger.ac.uk/cosmic.

[24] di Magliano MP, Logsdon CD (2013). Roles for KRAS in pancreatic tumor development and progression. Gastroenterology.

[25] Hruban RH, Goggins M, Parsons J, Kern SE (2000). Progression model for pancreatic cancer. Clinical cancer research : an official journal of the American Association for Cancer Research.

[26] Bodoky G, Timcheva C, Spigel DR, La Stella PJ, Ciuleanu TE, Pover G, Tebbutt NC (2012). A phase II open-label randomized study to assess the efficacy and safety of selumetinib (AZD6244 [ARRY-142886]) versus capecitabine in patients with advanced or metastatic pancreatic cancer who have failed first-line gemcitabine therapy. Investigational new drugs.

[27] Infante JR, Fecher LA, Falchook GS, Nallapareddy S, Gordon MS, Becerra C, DeMarini DJ, Cox DS, Xu Y, Morris SR, Peddareddigari VG, Le NT, Hart L, Bendell JC, Eckhardt G, Kurzrock R (2012). Safety, pharmacokinetic, pharmacodynamic, and efficacy data for the oral MEK inhibitor trametinib: a phase 1 dose-escalation trial. The lancet oncology.

[28] Infante JR, Somer BG, Park JO, Li CP, Scheulen ME, Kasubhai SM, Oh DY, Liu Y, Redhu S, Steplewski K, Le N (2014). A randomised, double-blind, placebo-controlled trial of trametinib, an oral MEK inhibitor, in combination with gemcitabine for patients with untreated metastatic adenocarcinoma of the pancreas. European journal of cancer.

[29] Hirai H, Iwasawa Y, Okada M, Arai T, Nishibata T, Kobayashi M, Kimura T, Kaneko N, Ohtani J, Yamanaka K, Itadani H, Takahashi-Suzuki I, Fukasawa K, Oki H, Nambu T, Jiang J (2009). Small-molecule inhibition of Wee1 kinase by MK-1775 selectively sensitizes p53-deficient tumor cells to DNA-damaging agents. Molecular cancer therapeutics.

[30] Said R, Hong DS, Warneke CL, Lee JJ, Wheler JJ, Janku F, Naing A, Falchook GS, Fu S, Piha-Paul S, Tsimberidou AM, Kurzrock R (2013). P53 mutations in advanced cancers: clinical characteristics, outcomes, and correlation between progression-free survival and bevacizumab-containing therapy. Oncotarget.

[31] Wade M, Li YC, Wahl GM (2013). MDM2, MDMX and p53 in oncogenesis and cancer therapy. Nature reviews Cancer.

[32] Tovar C, Graves B, Packman K, Filipovic Z, Higgins B, Xia M, Tardell C, Garrido R, Lee E, Kolinsky K, To KH, Linn M, Podlaski F, Wovkulich P, Vu B, Vassilev LT (2013). MDM2 small-molecule antagonist RG7112 activates p53 signaling and regresses human tumors in preclinical cancer models. Cancer research.

[33] ClinicalTrials.gov http://www.clinicaltrials.gov.

[34] Ezhevsky SA, Nagahara H, Vocero-Akbani AM, Gius DR, Wei MC, Dowdy SF (1997). Hypo-phosphorylation of the retinoblastoma protein (pRb) by cyclin D:Cdk4/6 complexes results in active pRb. Proceedings of the National Academy of Sciences of the United States of America.

[35] Wilentz RE, Geradts J, Maynard R, Offerhaus GJ, Kang M, Goggins M, Yeo CJ, Kern SE, Hruban RH (1998). Inactivation of the p16 (INK4A) tumor-suppressor gene in pancreatic duct lesions: loss of intranuclear expression. Cancer research.

[36] Liu F, Korc M (2012). Cdk4/6 inhibition induces epithelial-mesenchymal transition and enhances invasiveness in pancreatic cancer cells. Molecular cancer therapeutics.

[37] Chang DL, Qiu W, Ying H, Zhang Y, Chen CY, Xiao ZX (2007). ARF promotes accumulation of retinoblastoma protein through inhibition of MDM2. Oncogene.

[38] Blackford A, Serrano OK, Wolfgang CL, Parmigiani G, Jones S, Zhang X, Parsons DW, Lin JC, Leary RJ, Eshleman JR, Goggins M, Jaffee EM, Iacobuzio-Donahue CA, Maitra A, Cameron JL, Olino K (2009). SMAD4 gene mutations are associated with poor prognosis in pancreatic cancer. Clinical cancer research : an official journal of the American Association for Cancer Research.

[39] Iacobuzio-Donahue CA, Fu B, Yachida S, Luo M, Abe H, Henderson CM, Vilardell F, Wang Z, Keller JW, Banerjee P, Herman JM, Cameron JL, Yeo CJ, Halushka MK, Eshleman JR, Raben M (2009). DPC4 gene status of the primary carcinoma correlates with patterns of failure in patients with pancreatic cancer. Journal of clinical oncology : official journal of the American Society of Clinical Oncology.

[40] Singh P, Wig JD, Srinivasan R (2011). The Smad family and its role in pancreatic cancer. Indian journal of cancer.

[41] Levy L, Hill CS (2005). Smad4 dependency defines two classes of transforming growth factor {beta} (TGF-{beta}) target genes and distinguishes TGF-{beta}-induced epithelial-mesenchymal transition from its antiproliferative and migratory responses. Molecular and cellular biology.

[42] Guan B, Wang TL, Shih Ie M (2011). ARID1A, a factor that promotes formation of SWI/SNF-mediated chromatin remodeling, is a tumor suppressor in gynecologic cancers. Cancer research.

[43] Zeng Y, Liu Z, Yang J, Liu Y, Huo L, Li Z, Lan S, Wu J, Chen X, Yang K, Li C, Li M, Liu J (2013). ARID1A is a tumour suppressor and inhibits glioma cell proliferation via the PI3K pathwa. Head & neck oncology.

[44] Murphy KM, Brune KA, Griffin C, Sollenberger JE, Petersen GM, Bansal R, Hruban RH, Kern SE (2002). Evaluation of candidate genes MAP2K4, MADH4, ACVR1B, and BRCA2 in familial pancreatic cancer: deleterious BRCA2 mutations in 17%. Cancer research.

[45] Jones S, Hruban RH, Kamiyama M, Borges M, Zhang X, Parsons DW, Lin JC, Palmisano E, Brune K, Jaffee EM, Iacobuzio-Donahue CA, Maitra A, Parmigiani G, Kern SE, Velculescu VE, Kinzler KW (2009). Exomic sequencing identifies PALB2 as a pancreatic cancer susceptibility gene. Science.

[46] Villarroel MC, Rajeshkumar NV, Garrido-Laguna I, De Jesus-Acosta A, Jones S, Maitra A, Hruban RH, Eshleman JR, Klein A, Laheru D, Donehower R, Hidalgo M (2011). Personalizing cancer treatment in the age of global genomic analyses: PALB2 gene mutations and the response to DNA damaging agents in pancreatic cancer. Molecular cancer therapeutics.

[47] Chalasani P, Kurtin S, Dragovich T (2008). Response to a third-line mitomycin C (MMC)-based chemotherapy in a patient with metastatic pancreatic adenocarcinoma carrying germline BRCA2 mutation. JOP : Journal of the pancreas.

[48] Fong PC, Boss DS, Yap TA, Tutt A, Wu P, Mergui-Roelvink M, Mortimer P, Swaisland H, Lau A, O'Connor MJ, Ashworth A, Carmichael J, Kaye SB, Schellens JH, de Bono JS (2009). Inhibition of poly(ADP-ribose) polymerase in tumors from BRCA mutation carriers. The New England journal of medicine.

[49] Hezel AF, Gurumurthy S, Granot Z, Swisa A, Chu GC, Bailey G, Dor Y, Bardeesy N, Depinho RA (2008). Pancreatic LKB1 deletion leads to acinar polarity defects and cystic neoplasms. Molecular and cellular biology.

[50] Klumpen HJ, Queiroz KC, Spek CA, van Noesel CJ, Brink HC, de Leng WW, de Wilde RF, Mathus-Vliegen EM, Offerhaus GJ, Alleman MA, Westermann AM, Richel DJ (2011). mTOR inhibitor treatment of pancreatic cancer in a patient With Peutz-Jeghers syndrome. Journal of clinical oncology : official journal of the American Society of Clinical Oncology.

[51] Yokobori T, Yokoyama Y, Mogi A, Endoh H, Altan B, Kosaka T, Yamaki E, Yajima T, Tomizawa K, Azuma Y, Onozato R, Miyazaki T, Tanaka S, Kuwano H (2014). FBXW7 Mediates Chemotherapeutic Sensitivity and Prognosis in NSCLCs. Molecular cancer research : MCR.

[52] Solomona T, Racheta B, Whitehead S, Colemana M (2013). Cancer Survival in England: Patients Diagnosed 2007–2011 and Followed up to 2012.

[53] Wheler JJ, Lee JJ, Kurzrock R (2014). Unique Molecular Landscapes in Cancer: Implications for Individualized, Curated Drug Combinations. Cancer research.

[54] Liang WS, Craig DW, Carpten J, Borad MJ, Demeure MJ, Weiss GJ, Izatt T, Sinari S, Christoforides A, Aldrich J, Kurdoglu A, Barrett M, Phillips L, Benson H, Tembe W, Braggio E (2012). Genome-wide characterization of pancreatic adenocarcinoma patients using next generation sequencing. PloS one.

[55] Regine WF, Winter KA, Abrams R, Safran H, Hoffman JP, Konski A, Benson AB, Macdonald JS, Rich TA, Willett CG (2011). Fluorouracil-based chemoradiation with either gemcitabine or fluorouracil chemotherapy after resection of pancreatic adenocarcinoma: 5-year analysis of the U.S. Intergroup/RTOG 9704 phase III trial. Annals of surgical oncology.

[56] Oettle H, Post S, Neuhaus P, Gellert K, Langrehr J, Ridwelski K, Schramm H, Fahlke J, Zuelke C, Burkart C, Gutberlet K, Kettner E, Schmalenberg H, Weigang-Koehler K, Bechstein WO, Niedergethmann M (2007). Adjuvant chemotherapy with gemcitabine vs observation in patients undergoing curative-intent resection of pancreatic cancer: a randomized controlled trial. JAMA : the journal of the American Medical Association.

[57] Huguet F, Andre T, Hammel P, Artru P, Balosso J, Selle F, Deniaud-Alexandre E, Ruszniewski P, Touboul E, Labianca R, de Gramont A, Louvet C (2007). Impact of chemoradiotherapy after disease control with chemotherapy in locally advanced pancreatic adenocarcinoma in GERCOR phase II and III studies. Journal of clinical oncology : official journal of the American Society of Clinical Oncology.

[58] Komoto M, Nakata B, Amano R, Yamada N, Yashiro M, Ohira M, Wakasa K, Hirakawa K (2009). HER2 overexpression correlates with survival after curative resection of pancreatic cancer. Cancer science.

[59] Stoecklein NH, Luebke AM, Erbersdobler A, Knoefel WT, Schraut W, Verde PE, Stern F, Scheunemann P, Peiper M, Eisenberger CF, Izbicki JR, Klein CA, Hosch SB (2004). Copy number of chromosome 17 but not HER2 amplification predicts clinical outcome of patients with pancreatic ductal adenocarcinoma. Journal of clinical oncology : official journal of the American Society of Clinical Oncology.

[60] Harder J, Ihorst G, Heinemann V, Hofheinz R, Moehler M, Buechler P, Kloeppel G, Rocken C, Bitzer M, Boeck S, Endlicher E, Reinacher-Schick A, Schmoor C, Geissler M (2012). Multicentre phase II trial of trastuzumab and capecitabine in patients with HER2 overexpressing metastatic pancreatic cancer. British journal of cancer.

[61] Infante JR, Papadopoulos KP, Bendell JC, Patnaik A, Burris HA, Rasco D, Jones SF, Smith L, Cox DS, Durante M, Bellew KM, Park JJ, Le NT, Tolcher AW (2013). A phase 1b study of trametinib, an oral Mitogen-activated protein kinase kinase (MEK) inhibitor, in combination with gemcitabine in advanced solid tumours. European journal of cancer.

[62] LoRusso P, Shapiro G, Pandya SS, Kwak EL, Jones C, Belvin M, Musib LC, de Crespigny A, McKenzie M, Gates MR, Chan IT-C, Bendell JC (2012). A first-in-human phase Ib study to evaluate the MEK inhibitor GDC-0973, combined with the pan-PI3K inhibitor GDC-0941, in patients with advanced solid tumors. ASCO Meeting Abstracts.

[63] Rinehart J, Adjei AA, Lorusso PM, Waterhouse D, Hecht JR, Natale RB, Hamid O, Varterasian M, Asbury P, Kaldjian EP, Gulyas S, Mitchell DY, Herrera R, Sebolt-Leopold JS, Meyer MB (2004). Multicenter phase II study of the oral MEK inhibitor, CI-1040, in patients with advanced non-small-cell lung, breast, colon, and pancreatic cancer. Journal of clinical oncology : official journal of the American Society of Clinical Oncology.

[64] Lorusso PM, Adjei AA, Varterasian M, Gadgeel S, Reid J, Mitchell DY, Hanson L, DeLuca P, Bruzek L, Piens J, Asbury P, Van Becelaere K, Herrera R, Sebolt-Leopold J, Meyer MB (2005). Phase I and pharmacodynamic study of the oral MEK inhibitor CI-1040 in patients with advanced malignancies. Journal of clinical oncology : official journal of the American Society of Clinical Oncology.

